# State-Level Organization of Spontaneous Behavior and Its Association with Plasma Fatty-Acid Profiles in Healthy Rats

**DOI:** 10.3390/biology15080619

**Published:** 2026-04-16

**Authors:** Ahmed S. A. Ali Agha, Sara Khaleel, Nidal A. Qinna, Muhammed Alzweiri, Ghayda’ AlDabet, Thaqif El Khassawna, Talal Aburjai

**Affiliations:** 1 Department of Pharmaceutical Sciences, School of Pharmacy, The University of Jordan, Amman 11942, Jordan; ahm9220505@ju.edu.jo (A.S.A.A.A.); m.alzweiri@ju.edu.jo (M.A.); thaqif.elkhassawna@chiru.med.uni-giessen.de (T.E.K.); 2 Department of Pharmacy, Faculty of Pharmacy, Al-Zaytoonah University of Jordan, Amman 11733, Jordan; s.malkawi@zuj.edu.jo; 3 Faculty of Pharmacy and Medical Sciences, University of Petra, Amman 11196, Jordan; nqinna@uop.edu.jo; 4 University of Petra Pharmaceutical Center (UPPC), Amman 11196, Jordan; ghayda.aldabet@uop.edu.jo; 5 Experimental Trauma Surgery, Faculty of Medicine, Justus-Liebig-University of Giessen, 35392 Giessen, Germany

**Keywords:** behavioral variability, spontaneous behavior, exploratory behavior, grooming behavior, behavioral phenotyping, plasma lipid composition, fatty-acid profiling, multivariate analysis, correlation structure

## Abstract

Individual variability in spontaneous behavior emerges from coordinated physiological states, yet the organization of baseline behavioral dimensions and their systemic metabolic correlations remains insufficiently characterized in healthy animals. Here, exploratory activity and self-directed grooming were quantified during a standardized Y-maze session and integrated with plasma fatty-acid profiles measured by orthogonal dual-column GC-MS in 30 healthy male Sprague–Dawley rats distributed across four independent cohorts. Correlation structure and principal component analysis showed that plasma lipid profiles exhibit non-random, low-dimensional organization, resolving into coherent compositional states dominated by coordinated shifts between shorter-chain (C14–C16) and longer-chain (C18) fatty-acid species. Behavioral variables are similarly organized into constrained dimensions capturing exploratory engagement and grooming expression or timing. Integrated correlation analysis indicated that plasma fatty-acid-derived lipid states aligned systematically with behavioral organization at the multivariate level across cohorts, with C18-enriched profiles generally aligned with higher behavioral engagement and earlier grooming expression at the state level, while C14/C16-enriched profiles associated with reduced exploration and delayed grooming initiation, although the direction of individual lipid–behavior correlations showed cohort dependence consistent with the context-dependent behavior of compositional lipid variables within distinct but physiologically normal lipidomic states. These findings demonstrate that state-level, correlation-based multivariate analysis can reveal structured plasma lipidomic configurations and their correspondence to behavioral organization under physiological conditions, without inferring causal directionality or pathway activation.

## Simple Summary

Animals naturally differ in how actively they explore their environment and how they engage in self-directed behaviors such as grooming, even when they are healthy and kept under similar conditions. Understanding the biological basis of this normal variation is important for interpreting behavior in research studies. In this work, we examined whether patterns of spontaneous behavior are linked to overall physiological organization in healthy laboratory rats. Rats were observed during a standardized short exploration task, and their movement and grooming behaviors were recorded. Blood samples were then analyzed to measure different types of fatty acids, which reflect how fats are processed in the body. We found that both behavior and blood fatty-acid composition were organized into clear patterns rather than varying randomly between individuals. Importantly, these physiological patterns showed consistent alignment with behavioral organization across independent groups of healthy animals. Rather than aiming to predict behavior in individual animals, our findings outline a structured framework that can support the comparison and interpretation of behavioral and physiological variation in future studies of healthy populations. This approach helps researchers better understand normal biological variability and supports more consistent interpretation of behavioral data in biological and biomedical research.

## 1. Introduction

Lipids are essential to life, serving not only as structural components of cellular membranes but also as dynamic regulators of energy homeostasis, signaling, and intercellular communication [1,2]. The plasma lipidome—comprising hundreds of distinct lipid molecular species—acts as an integrated reflection of systemic metabolic activity and environmental influences [3]. Importantly, systemic metabolic organization can manifest in organismal phenotypes, including spontaneous behavioral tendencies that reflect arousal, engagement, and state regulation of coping [4]. Despite clear evidence that baseline plasma lipid composition varies systematically with physiological factors such as feeding state, sex, and age [5,6], foundational work remains limited in two respects. The pathway-level determinants of baseline plasma fatty-acid architecture in healthy rodents—spanning desaturation, elongation, and remodeling—are under characterized outside disease or under conditions of strong experimental perturbations. Second, it remains unclear whether naturally occurring lipidomic configurations correspond to reproducible dimensions of spontaneous behavior within normative populations, rather than reflecting analytically isolated lipid differences.

From a systems-biology and behavioral-phenotyping perspective, this gap reflects a shortage of analytical frameworks designed to resolve structured, multivariate organization within baseline plasma lipid composition data and to relate that organization to low-dimensional behavioral structure, rather than focusing on isolated lipid-level differences. Among the analytical technologies, gas chromatography-mass spectrometry (GC-MS) retains a unique position for quantitative characterization of fatty acid methyl esters (FAMEs), offering high chromatographic resolution, reproducibility, and extensive spectral libraries for compound identification [7,8]. In behavioral–physiological integration studies, such interpretability is valuable because fatty-acid features provide a pathway-linked readout of systemic lipid handling that can be compared across independent cohorts and related to organism-level behavioral phenotypes.

However, one-dimensional GC-MS plasma fatty-acid profiling can be hindered by matrix effects and methodological heterogeneity, including derivatization-dependent variability and baseline distortions that obscure minor yet biologically relevant species [9,10].

In addition to analytical constraints, data interpretation represents a critical bottleneck in lipidomics. This issue is particularly relevant for plasma fatty-acid profiles because lipidomic data are inherently compositional; each lipid is measured as part of a constrained total and therefore varies in relation to all other components rather than independently. As emphasized by Quinn et al. (2019), such data should be interpreted in terms of coordinated multivariate organization rather than isolated pairwise changes [11], and Greenacre et al. (2021) further showed that the apparent direction and strength of individual associations can vary across datasets depending on the underlying covariance structure and reference composition [12]. This makes state-level and correlation-structure-based analyses especially appropriate for resolving reproducible biological organization in baseline lipidomic datasets.

These limitations motivate robust, standardized workflows, and complementary multivariate analyses to distinguish coherent biological co-variation from analytical noise. In addition to analytical constraints, data interpretation represents a critical bottleneck in lipidomics. Conventional univariate or isolated pairwise approaches capture only limited aspects of interdependent metabolic regulation [13,14], and are similarly mismatched to behavioral phenotypes that typically emerge as low-dimensional combinations of correlated measures. In this context, chemometric and multivariate approaches—such as principal component analysis (PCA), hierarchical clustering, and heat-map visualization—provide robust frameworks for revealing the latent structure, quantifying reproducibility, and mapping metabolic states onto behavioral organization [15,16].

Beyond its metabolic and structural roles, the lipidome interfaces with brain function and behavior through contributions to neuronal membrane composition, excitability, and neuroenergetic coupling, and peripheral lipid composition has been associated with individual differences in cognition and stress responsivity [17,18]. However, most lipid–behavior studies have focused on disease, pharmacological, or dietary manipulations, leaving the intrinsic organization of baseline plasma lipidomic states and their correspondence to spontaneous behavioral dimensions in healthy animals largely unexplored.

Previous studies examining links between lipid metabolism and behavior have predominantly focused on isolated lipid species or on lipidomic alterations induced by disease states, dietary interventions, or pharmacological manipulation [19,20,21,22]. While such approaches are valuable for identifying condition-specific biomarkers, they do not resolve how baseline plasma lipid composition is organized as a coordinated multivariate system under physiological conditions, nor how such organization aligns with spontaneous behavioral variability in healthy populations. The present work departs from univariate and perturbation-driven paradigms by adopting a correlation–structure-based, state-level analytical framework. Rather than identifying individual lipid predictors of behavior, this approach demonstrates that reproducible modular lipidomic states align systematically with low-dimensional behavioral organization across independent healthy cohorts.

In this study, we combined orthogonal dual-column GC-MS fatty-acid profiling with standardized behavioral assessment and multivariate correlation-structure analysis to characterize baseline plasma lipidomic organization and its correspondence to naturally occurring behavioral variation in healthy male Sprague–Dawley rats across four independent cohorts. By mapping lipidomic covariance modules onto low-dimensional behavioral structure, this work provides an empirical, state-level framework for relating systemic lipid organization to behavior under physiological conditions, while explicitly avoiding causal inference or claims of pathway activation.

This framing is especially important for compositional lipid datasets, where biological interpretation is more robust at the level of coordinated states and covariance structure than at the level of isolated lipid–behavior pairings.

## 2. Materials and Methods

### 2.1. Chemicals and Reagents

Methanol (HiPerSolv CHROMANORM, HPLC gradient grade, VWR Chemicals, Fontenay-Sous-Bois, France); potassium hydroxide pellets (≥85%, Analytical Reagent grade, AR); boron trifluoride in methanol (12% *w*/*w* BF_3_, 1.5 M, AcroSeal^®^, Thermo Scientific, Waltham, MA, USA); sulfuric acid (95–97%, J.T. Baker, Mallinckrodt Baker B.V., Deventer, The Netherlands); n-hexane (high-purity “PESTIPUR” grade, Carlo Erba Reagents S.A.S., Val de Reuil, France); iso-octane (GC grade, CAS 540-84-1; Bahadurgarh, Haryana, India); dimethyl azelate (technical grade, 80%, Aldrich Chemistry, Sigma-Aldrich, St. Louis, MO, USA) used as the internal standard. A 37-component FAME reference mixture (FAME-37, Supelco; Cat. No. 47885-U). Helium (≥99.999%, with O_2_/H_2_O traps) served as the carrier gas; nitrogen (≥99.999%) was used for evaporation.

### 2.2. Animals and Housing

Male Sprague–Dawley rats (average body weight ≈ 255 ± 10 g at the start of the experiment) were obtained and housed in the animal facility of the University of Petra (Amman, Jordan). Animals were acclimatized for at least 10 days before experimentation. Rats were maintained under controlled environmental conditions (temperature 22–24 °C, relative humidity 55–65%) on a 12 h light/12 h dark cycle with free access to standard chow and water. Animals were group-housed within each cohort (*n* = 7–8 per cohort), with all animals from the same cohort maintained in a single cage under identical environmental conditions. All procedures complied with the institutional guidelines for the care and use of laboratory animals and with the principles of the Federation of European Laboratory Animal Science Associations (FELASA). The experimental protocol was approved by the local Institutional Animal Care and Use Committee at the University of Petra (IACUC Protocol Number: E/A/7/11/2025). Reporting followed the ARRIVE guidelines; the completed checklist is provided as a separate file.

Given the established influence of gut microbiota on fatty-acid metabolism, microbiome-related variability was actively controlled during study design. A bedding-transfer procedure was implemented across cages during the pre-experimental period to promote microbiome homogenization, based on established normalization strategies such as those described by Miyoshi et al. (2018) [23], in which the redistribution of soiled bedding reduces inter-individual and cage-associated microbiome variability. In parallel, all animals were maintained under identical dietary and environmental conditions and matched for strain, age, and sex to control additional determinants of microbiome composition. Together, these measures were applied to minimize baseline microbiome heterogeneity and reduce its potential contribution as a confounding factor in the observed lipidomic organization.

**Figure 1:**
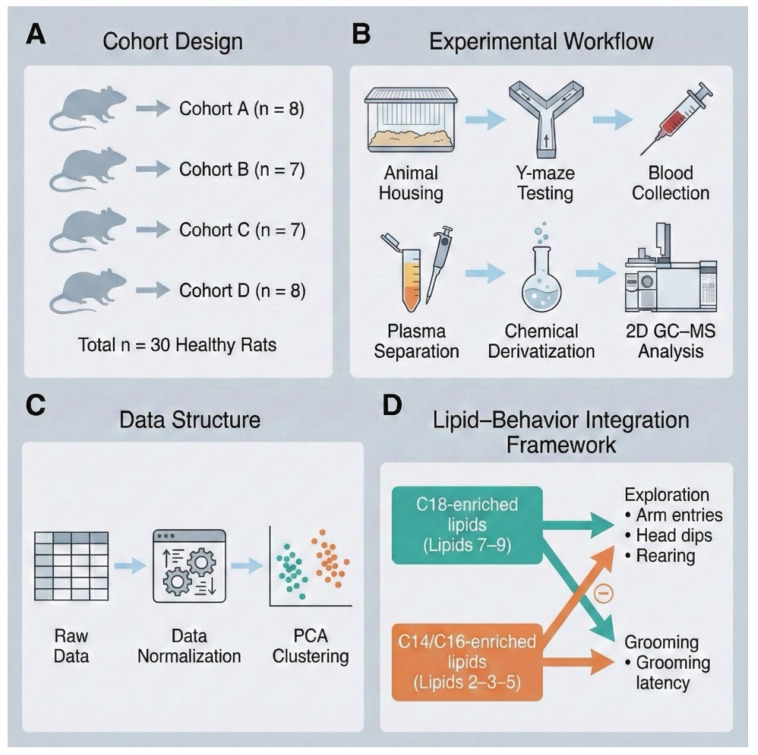
Overview of study design and lipid–behavior integration. (**A**) Allocation of 30 healthy rats into four cohorts (**A**–**D**). (**B**) Experimental workflow from animal housing and Y-maze testing to blood collection, plasma separation, chemical derivatization, and orthogonal dual-column GC-MS lipid analysis. (**C**) Data processing pipeline including normalization and PCA-based clustering. (**D**) Integrative framework linking C18-enriched lipids (Lipids 7–9) with exploratory behaviors and C14/C16-enriched lipids (Lipids 2–3–5) with grooming-related measures, illustrating coordinated lipid–behavior associations.

Four independent cohorts of healthy rats were used. Cohort A comprised eight rats (A1–A8), Cohort B comprised seven rats (B1–B7), Cohort C comprised seven rats (C1–C7), and Cohort D comprised eight rats (D1–D8). All animals underwent identical behavioral testing and were subsequently sampled for plasma lipidomic analysis. No pharmacological, dietary, or disease-inducing interventions were applied; the study was designed to characterize naturally occurring inter-individual variation in lipidomic and behavioral organization across independent healthy cohorts. An overview of the cohort structure, experimental workflow, and analytical framework is presented in Figure 1.

### 2.3. Y-Maze Apparatus and General Behavioral Procedures

Exploratory behavior and self-directed grooming were assessed in a Y-maze apparatus, adapted from a standard spontaneous alternation paradigm to quantify locomotor and investigatory activity. The maze was constructed from wood and consisted of three identical arms (75 cm long × 15 cm wide) with 10 cm high walls. The arms were arranged at 120° angles to form a symmetrical Y-shaped configuration. The maze was placed in a quiet, evenly lit room.

Behavioral testing was conducted at least 24 h after the last handling session to minimize acute handling-related arousal effects. All behavioral observations were conducted during the daytime within a consistent time window across all animals.

At the start of each session, the rat was placed at the center of the maze facing the same arm orientation. Each animal was allowed to explore freely for 5 min. No external cues, baits, or rewards were introduced, thereby relying on the animals’ intrinsic exploratory drive. The maze was cleaned and dried between trials to minimize olfactory cues.

### 2.4. Behavioral Scoring

Behavioral variables were scored by observers blinded to the lipidomic data. During the 5-min Y-maze trial, the number of arm entries was recorded as the total number of times each rat entered an arm, head-dip behavior was quantified as the number of occasions on which the animal extended its head over the distal end or into the corners of the arms as an index of investigatory activity, and rearing was measured as the number of vertical exploratory events in which the rat stood on its hindlimbs, with or without wall support. Self-directed behavior was assessed by recording the total number of discrete grooming bouts, including face-washing and body grooming, and by measuring the latency to first grooming, defined as the time in seconds from placement in the maze center until the onset of the first grooming episode. These measures were selected to capture complementary behavioral domains, with arm entries, head dips, and rearing reflecting locomotor and investigatory exploration, and grooming frequency and grooming latency reflecting self-directed and affective behavioral components. Raw behavioral measurements for each individual animal across all cohorts are provided in Supplementary Tables S1A–S1D.

### 2.5. Blood Collection and Plasma Preparation

Immediately after the completion of behavioral testing, rats were anesthetized with isoflurane (induction ~5%, maintenance ~2.5% in 0.5 L/min oxygen) using a low-flow anesthesia system to minimize handling-related stress during blood collection, as acute sampling stress—including that associated with tail-based methods—has been known to induce rapid alterations in circulating metabolites [24]; blood was then collected from the retro-orbital sinus using heparinized capillary tubes into EDTA-coated microtubes (Hangzhou Kimislab Co., Ltd., Hangzhou, China).

Samples were centrifuged at 6000× *g* for 10 min at 4 °C to separate plasma. The plasma fraction was carefully transferred to clean tubes, aliquoted to avoid repeated freeze–thaw cycles, and stored at −80 °C until lipidomic analysis. All procedures followed local ethical requirements.

### 2.6. Plasma Lipidomics by Orthogonal Dual-Column GC-MS

A targeted fatty-acid-profiling lipidomics workflow was applied to all plasma samples from the four cohorts. Although this approach profiles fatty-acid-derived features rather than intact lipid classes, it provides a chemically interpretable, pathway-linked readout of systemic lipid metabolism suitable for cohort-level modular analyses. The method combines an alkaline–acidic derivatization sequence with two serially coupled capillary columns providing orthogonal chromatographic selectivity in a dual-column GC-MS configuration to ensure consistent identification and quantification of fatty acid methyl esters (FAMEs). Frozen plasma aliquots were thawed on ice and vortexed briefly. Lipids were first saponified by adding 0.5 M KOH in methanol and heating at 80 °C for 10 min to release fatty acids from esterified lipid species. After cooling, concentrated sulfuric acid was added to neutralize the mixture and halt the alkaline reaction. Fatty acids were extracted into n-hexane by liquid–liquid partitioning; the organic layer was collected and evaporated to dryness under a gentle nitrogen stream to prevent oxidation.

The dried extract was reconstituted in boron-trifluoride–methanol reagent (≈12% BF_3_, 1.5 M) to complete transmethylation. The resulting FAMEs were transferred to amber GC vials and injected immediately. A fixed volume of dimethyl azelate was added before GC-MS injection as an internal standard to correct for injection variability. Each plasma sample was analyzed once (technical replicate *n* = 1), with biological replication provided by independent animals within each cohort.

It should be noted that the present analytical approach quantifies fatty-acid composition rather than enzyme activity or metabolic flux. Accordingly, references to lipid-metabolic processes such as desaturation (e.g., SCD1), elongation (ELOVL), or polyunsaturated fatty-acid metabolism (FADS1/2) were not directly measured in this study. Any pathway-level interpretation is therefore derived from compositional relationships among fatty-acid species within the constraints of plasma fatty-acid profiling. All cohorts were processed using an identical experimental workflow, including standardized behavioral testing, uniform derivatization procedures, and the same GC-MS analytical configuration, with internal standard normalization applied to minimize technical variability.

#### 2.6.1. GC-MS Instrumentation

FAMEs were analyzed using a GC-MS system equipped with two capillary columns connected in series to provide orthogonal selectivity. The first column consisted of a polar polyethylene-glycol phase (TRB-Wax Ω, 20 m × 0.125 mm, 0.125 µm), followed by a second low-polarity column (SCION-5MS, 30 m × 0.25 mm, 0.25 µm). Helium carrier gas was maintained at ~1.0 mL/min. Samples (1 µL) were injected in on-column mode, and the injector was held at 250 °C.

The oven program began at 100 °C (1 min), ramped at 10 °C/min to 250 °C, and was held for ~40 min (total runtime ≈ 56 min), enabling full separation across short-, medium-, and long-chain FAMEs. The MS operated in electron ionization mode (EI, 70 eV) with data acquired in centroid mode over *m*/*z* 40–500. A solvent delay (~6 min) was applied to prevent early-eluting solvent contamination.

#### 2.6.2. Identification and Selection of Lipid Variables

Identification of individual FAMEs was achieved using a combined retention-time and mass-spectral approach. First, each analyte was matched to the NIST EI mass-spectral libraries (minimum match factor ≥ 700), providing primary spectral confirmation. To ensure chromatographic reliability, all experimental retention times were additionally compared with those obtained from the Supelco 37-component FAME standard (FAME-37) analyzed under identical dual-column conditions. Because retention behavior can differ across column geometries, the experimental elution order was further cross-checked qualitatively against an external reference chromatogram of the same FAME-37 mixture acquired on an Omegawax 250 column (courtesy of Prof. Luigi Mondello, University of Messina; reference chromatogram distributed by Supelco, Bellefonte, PA, USA). Agreement in relative retention sequence across both the in-house dual-column system (TRB-Wax Ω → SCION-5MS) and the Omegawax reference ensured orthogonal confirmation of chain-length and unsaturation ordering, providing high confidence in FAME identity assignments.

To further examine retention behavior in the C14–C16 region, selected samples were additionally analyzed by GC-FID under comparable chromatographic conditions as a complementary detection approach, providing independent confirmation of retention characteristics.

### 2.7. Lipid Selection and Data Structure

Raw GC-MS chromatograms consisted of approximately 3600 intensity values per sample, corresponding to the 0–60 min acquisition window. To standardize the data and reduce noise-driven variability, chromatograms were processed using a bin-sum normalization routine in which consecutive intensity values were aggregated into 3-s bins. This procedure reduced each chromatogram to 1200 bins while preserving retention-time structure and minimizing injection-volume effects. The binned intensities were subsequently normalized to the total ion current (TIC) of each sample, converting the chromatograms from intensity-driven to composition-driven profiles suitable for chemometric analysis.

Peak areas (AUCs) for all detectable fatty acid methyl esters were then computed directly from the normalized chromatograms. From these, nine lipid species were consistently resolved across all animals based on peak shape, spectral match quality, and biological relevance (chain length and degree of unsaturation). These species were designated Lipid 1–Lipid 9 for statistical modeling. For each animal, the area of each lipid peak was expressed as the proportional contribution of that peak to the sum of AUCs of all nine identified lipids, yielding a 9-dimensional compositional vector that summed to 100%.

Cohort A consisted of eight rats (A1–A8) and yielded an 8 × 9 lipid matrix; Cohort B consisted of seven rats (B1–B7) and yielded a 7 × 9 matrix; Cohort C consisted of seven rats (C1–C7) and yielded a 7 × 9 matrix; and Cohort D consisted of eight rats (D1–D8) and yielded an 8 × 9 matrix. Each cohort was analyzed independently, and where appropriate, in direct relation to its corresponding behavioral dataset to evaluate the consistency of lipidomic–behavioral organization across independent healthy cohorts.

### 2.8. Statistical Analysis and Data Visualization

Statistical analyses and primary visualizations were performed in R (version 4.5.1). Descriptive statistics, Pearson correlation analyses, and principal component analysis (PCA) were conducted using base R functions (including prcomp). Correlation analysis was used to characterize association structure and modular organization rather than to infer directional or causal relationships. Data manipulation was carried out using dplyr and tidyr. Correlation heatmaps were generated using ggplot2 in combination with reshape2 or tidyr for matrix reshaping; in selected instances, enhanced annotated heatmaps were produced using ComplexHeatmap for improved visualization. Scatterplots with regression lines were generated using ggplot2.

For each cohort, Pearson correlation coefficients (r) were computed among the nine lipid variables (lipid–lipid matrices), among the behavioral variables (behavior–behavior matrices), and between lipid and behavioral variables (combined lipid–behavior matrices). Given the modest sample sizes and the exploratory nature of the study, correlation results were interpreted primarily in terms of effect size, directionality, and coherence of modular patterns. Accordingly, effect size and structured covariance patterns were considered more informative than statistical significance for interpreting lipid–behavior relationships in this study.

Benjamini–Hochberg false discovery rate (FDR) correction was applied to all correlation matrices within each cohort, and adjusted *p*-values (q-values) are reported in Supplementary Tables S2–S4; however, interpretation focused on reproducible multivariate organization rather than individual pairwise significance.

For the behavioral variable latency to first grooming, some animals did not initiate grooming during the 5-min Y-maze session, resulting in missing values (N/A). To standardize these observations across cohorts and avoid variable sample sizes across correlation matrices, animals that did not groom during the observation period were assigned a latency value of 300 s, corresponding to the full duration of the test window. All behavior–behavior and lipid–behavior correlations were then recomputed using the completed dataset. This procedure produced only modest numerical adjustments and did not alter the overall multivariate structure or the principal cohort-level patterns. All other behavioral variables were complete for all animals within each cohort.

PCA was performed separately for each cohort (Cohorts A–D) using the nine lipid variables expressed as relative percentages. Prior to PCA, variables were mean-centered but not variance-scaled (i.e., center = TRUE, scale = FALSE), because all features were measured on a common percentage scale and preservation of relative compositional amplitude was desired. The first two principal components (PC1 and PC2) were used for visualization, and the proportion of variance explained by each component was reported. Unsupervised phenotype structure within each cohort was evaluated using k-means clustering (k = 3) applied to the PC1–PC2 score space, with cluster membership determined solely by the k-means algorithm. For graphical representation, 95% confidence ellipses were overlaid using ggplot2 (stat_ellipse, type = “norm”) to illustrate group dispersion; these ellipses did not influence clustering and served only as descriptive summaries of multivariate structure.

Behavioral correlation matrices were constructed separately for each cohort to identify low-dimensional behavioral organization (e.g., exploration-related and grooming-timing axes). Lipid–behavior relationships were visualized using heatmaps of the combined correlation matrices to assess alignment between the lipidomic modules and behavioral dimensions.

All analyses were pre-specified as exploratory and hypothesis-generating. The primary objective was to characterize modular organization within lipidomic and behavioral data spaces using correlation-based and multivariate analytical frameworks and to evaluate the consistency of lipid–behavior mappings across four independent healthy cohorts, rather than to establish causal or diagnostic claims.

## 3. Results and Discussion

### 3.1. Plasma Lipid Species Detected Across Cohorts

Across all four cohorts, nine fatty-acid-derived lipid species were consistently detected and quantified and were therefore retained for all subsequent analyses (Table 1).

To further evaluate retention behavior in the C14–C16 region, complementary GC-FID analysis was performed. This analysis indicated that peaks within this region are intrinsically closely spaced under the applied chromatographic conditions, consistent with the well-established elution proximity of structurally similar fatty-acid methyl esters, thereby limiting isomer-level resolution based on retention time. This observation supports the conservative assignment strategy adopted for Lipid 3 and Lipid 4.

Compound identities were assigned using EI mass-spectral matching against the NIST libraries, verified by retention behavior on the dual-column GC-MS system, and confirmed using the Supelco 37-component FAME reference mixture. The identified species span saturated, monounsaturated, and polyunsaturated fatty acids ranging from C13:0 to C18:3, enabling pathway-interpretable comparisons based on carbon-chain length and degree of unsaturation. For clarity, Lipids 1–9 correspond respectively to C13:0, C14:0, an unresolved feature in the C14 region, a putative C14:1-associated feature, C16:1 n-7, C17:1 n-7, C18:0, C18:1 n-9, and C18:3 n-6 (Table 1). Rather than treating these variables as isolated entities, subsequent analyses focused on their coordinated variation, which provides insight into the organization of the plasma lipidome under physiological conditions.

Tridecanoic acid (C13:0), an odd-chain fatty acid with distinct metabolic or microbial origins, was consistently detected and analytically confirmed across all samples. It was retained as part of the compositional lipidomic structure rather than interpreted individually, and did not influence the state-level organization, which was driven primarily by coordinated variation among C14–C18 species.

Importantly, the study’s central conclusions are not dependent on definitive structural assignments of individual peaks in this region. Rather, interpretation is based on the reproducible covariance structure and modular organization of lipid variables across independent cohorts, whereby the C14/C16-associated module remains robustly defined by the coordinated variation of neighboring lipid features.

### 3.2. Modular Organization of Plasma Lipid Composition

**Figure 2:**
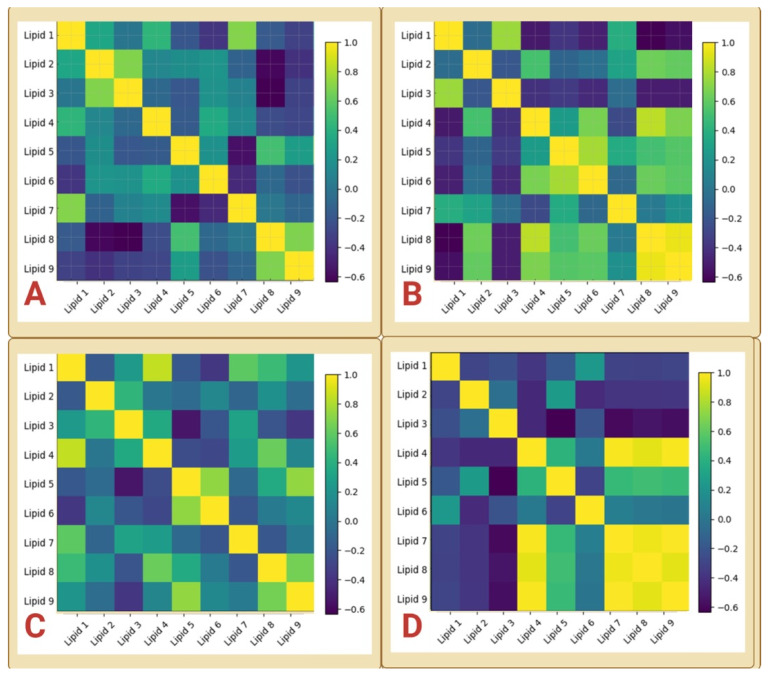
Lipid–lipid correlation structure across independent cohorts. Heatmaps show Pearson correlation matrices of the nine plasma lipid variables (Lipids 1–9) analyzed separately for Cohort A (**A**), Cohort B (**B**), Cohort C (**C**), and Cohort D (**D**). Each matrix represents pairwise correlations among lipid variables within a cohort, with color intensity indicating correlation strength and direction (yellow, positive; purple, negative). Across all cohorts, the matrices exhibited non-random, block-like patterns, indicating coordinated variation among subsets of lipids rather than independent fluctuation of individual species. While the overall modular organization was preserved across cohorts, the specific lipid combinations defining positively and negatively correlated blocks varied, reflecting cohort-dependent weighting of lipidomic relationships within a shared modular framework.

Lipid–lipid correlation matrices computed separately for Cohorts A–D revealed that plasma lipid composition varied in a structured rather than random manner in all cohorts (Figure 2A–D).

In each case, the matrices were characterized by contiguous regions of positive and negative correlations, indicating the presence of coordinated lipid subsets that vary together across animals. Although the exact composition of these subsets differed among cohorts, a recurring feature was the emergence of antagonistic relationships between longer-chain C18-associated lipids and subsets enriched in shorter-chain saturated or monounsaturated species. For example, in Cohorts A and D, Lipids 7–9 (C18 species) formed a tightly covarying block that opposed C14/C16-enriched components (Figure 2A,D), whereas in Cohorts B and C, the dominant opposing axes were anchored by different lipid combinations while preserving the same non-random, modular architecture (Figure 2B,C). These findings indicate that across independent healthy cohorts, the plasma lipidome consistently segregates into coordinated compositional states, even though the specific lipid variables that dominate each state may shift with metabolic context.

Within cohorts, correlations defining these lipid modules were typically moderate to strong (|r| ≈ 0.4–0.9), indicating tightly coordinated variation; however, consistent with FDR-adjusted analysis, these values were interpreted as components of multivariate structure rather than independent pairwise evidence. After Benjamini–Hochberg correction, no individual lipid–lipid correlations remained formally significant in Cohorts A or C, whereas only a limited subset of very strong correlations survived correction in Cohort B and a tightly coupled Lipid 4/7/8/9 module remained robustly significant in Cohort D. Thus, the corrected results indicate that pairwise statistical support varies substantially across cohorts, while the broader block-like organization of the plasma lipidome is preserved at the matrix level.

Complete lipid–lipid correlation matrices for all cohorts are provided in Supplementary Tables S2A–S2D.

### 3.3. Stratification of Plasma Lipid States Revealed by PCA

**Figure 3:**
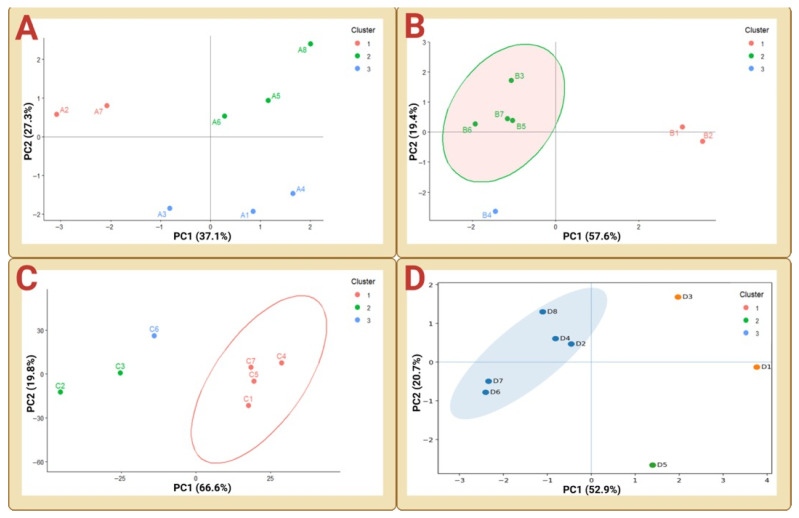
Principal component analysis (PCA) of plasma lipidomic profiles across independent cohorts. PCA was performed independently within each cohort using relative lipid composition values. Score plots of the nine plasma lipid variables are shown separately for Cohort A (**A**), Cohort B (**B**), Cohort C (**C**), and Cohort D (**D**). Each point represents an individual animal, labeled by cohort identifier, and colors denote unsupervised k-means cluster membership (k = 3) determined within each cohort. Axes correspond to the first two principal components, with the percentage of variance explained indicated in parentheses. Ellipses represent 95% confidence regions for each cluster and are included solely for visualization of group dispersion. Across cohorts, PCA resolves animals into discrete lipid-defined groupings, indicating structured, low-dimensional organization of plasma lipidomic variation within healthy rats, while the relative separation and orientation of clusters vary by cohort.

To determine whether the observed correlation structures corresponded to low-dimensional plasma lipid states, principal component analysis was applied independently to each cohort. In all cohorts, PCA resolved animals into discrete groupings in PC1–PC2 space, indicating that inter-individual lipidomic variability is organized around a limited number of compositional configurations rather than distributed continuously and confirming that lipidomic variation is structured at the multivariate level rather than driven by isolated lipid fluctuations (Figure 3A–D). To assess whether PCA structure was disproportionately influenced by differences in lipid variance, PCA was additionally performed on standardized lipid variables (centered and variance-scaled). This analysis yielded comparable clustering patterns (Figure S1), indicating that the observed groupings are not driven solely by high-variance or high-abundance lipid species.

In Cohort A, separation was driven primarily by opposition between a C18-enriched profile and a C14/C16-enriched profile, with one animal occupying an extreme position along the dominant axis (Figure 3A). Cohort B exhibited a similarly structured separation, including a distinct outlying profile relative to the remaining animals (Figure 3B), while Cohorts C and D resolved into phenotypic groupings consistent with their cohort-specific correlation patterns (Figure 3C,D), with Cohort C showing a modest change in cluster geometry upon variance scaling (Figure S1) while preserving overall group structure.

Across cohorts, PCA-defined groupings aligned with the major lipid–lipid covariance blocks identified within each cohort, indicating that lipidomic stratification reflects coordinated variation among lipid subsets rather than diffuse compositional shifts.

Across cohorts, the first two principal components together explained a substantial proportion of lipidomic variance (typically ~65–85%), indicating that inter-individual differences are concentrated along a limited number of compositional axes. In all cohorts, PC1 accounted for the majority of explained variance and was consistently driven by opposing contributions of C18-centered versus C14/C16-centered lipid species, identifying a dominant compositional axis underlying lipidomic stratification.

### 3.4. Low-Dimensional Behavioral Organization

**Figure 4:**
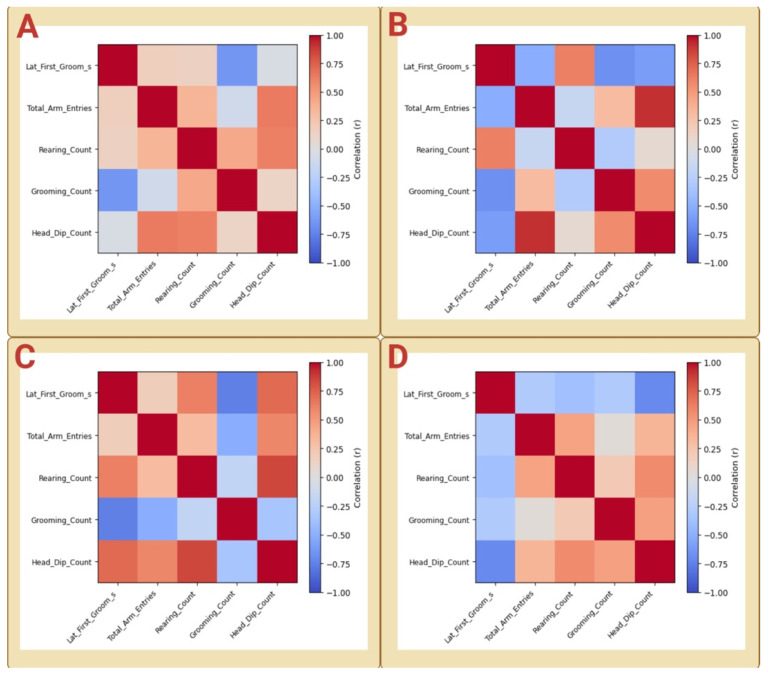
Behavioral correlation structure across independent cohorts. Heatmaps show Pearson correlation matrices among behavioral variables measured during the Y-maze task for Cohort A (**A**), Cohort B (**B**), Cohort C (**C**), and Cohort D (**D**). Variables include latency to first grooming, total arm entries, rearing count, grooming count, and head-dip count. Color intensity represents the strength and direction of the correlations (red, positive; blue, negative). Across cohorts, behavioral measures exhibit structured, non-random patterns of association, indicating low-dimensional organization of exploratory and grooming-related behaviors, while the relative alignment among measures varies across independent cohorts.

Behavioral variables likewise exhibited structured organization at the multivariate level across cohorts. Behavior–behavior correlation matrices revealed that exploratory measures and grooming-related measures clustered into coherent dimensions in all cohorts, although the relative weighting of individual behaviors varied (Figure 4A–D).

In Cohorts A and B, head-dip behavior showed consistent positive association with total arm entries, whereas rearing exhibited cohort-dependent integration, aligning with head-dip behavior in Cohort A but not in Cohort B. Grooming measures did not exhibit a consistent inverse relationship with exploratory variables (Figure 4A,B). In Cohort C, investigatory behaviors (head dips and rearing) remained tightly coupled, with arm entries showing moderate but comparatively weaker integration within this behavioral module (Figure 4C). In Cohort D, grooming frequency aligned positively with head-dip and rearing measures, indicating a broader engagement-related configuration rather than a strict exploration–grooming trade-off (Figure 4D). This pattern is consistent with the context-dependent nature of rodent grooming behavior. Grooming in rats can be elicited by novelty and does not have a single fixed behavioral meaning across test conditions, with its interpretation depending on the experimental context [25,26]. Experimental observations further indicate that grooming patterns change across successive bouts and vary across test situations, and that their interpretation differs across contexts rather than reflecting a single stable behavioral dimension [25,27]. Within the standardized conditions of the present study, the absence of a consistent exploration–grooming trade-off across cohorts can be understood as a shift in the behavioral relationships captured within the same test environment, rather than a consequence of uncontrolled variability or a fundamental change in the underlying behavioral organization. Accordingly, the positive alignment between grooming and investigatory activity observed in Cohort D is most appropriately interpreted as a context-dependent reorganization of behavioral structure within the same constrained behavioral space, rather than a loss of the underlying organization or a contradiction of the patterns observed in earlier cohorts.

Despite these cohort-specific differences, all cohorts displayed low-dimensional behavioral structure, indicating that spontaneous behavior in healthy rats occupies a constrained behavioral space rather than reflecting independent variation across measures.

Within cohorts, strongly coupled behavioral measures typically exhibited moderate-to-high correlations (|r| ≈ 0.4–0.9), indicating coordinated variation among behavioral variables. After Benjamini–Hochberg correction, formal pairwise support for individual behavioral associations was absent in Cohorts A, C, and D and limited in Cohort B, where arm entries and head-dip behavior remained strongly associated. These results indicate that the statistical robustness of individual behavioral pairings varies across cohorts, whereas the broader organization of exploratory and grooming-related measures is more consistently preserved at the matrix level.

Consistent with the FDR-adjusted analysis, these associations are interpreted as components of low-dimensional behavioral organization rather than as independent pairwise relationships. Complete behavior–behavior correlation matrices for all cohorts are provided in Supplementary Tables S3A–S3D.

### 3.5. Integration of Lipidomic and Behavioral Organization

**Figure 5:**
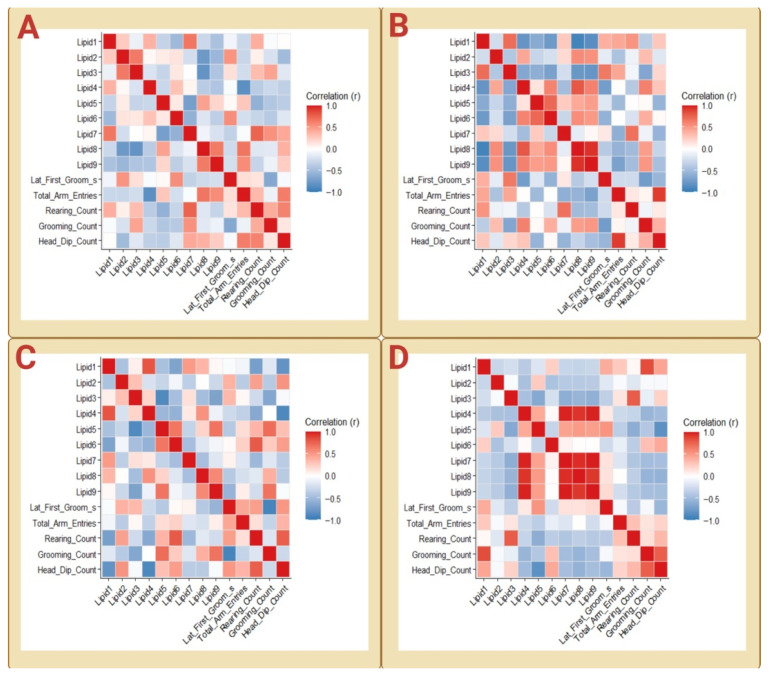
Integrated lipid–behavior correlation structure across independent cohorts. Heatmaps show Pearson correlation matrices combining plasma lipid variables (Lipids 1–9) and behavioral measures obtained during the Y-maze task for Cohort A (**A**), Cohort B (**B**), Cohort C (**C**), and Cohort D (**D**). Behavioral variables include latency to first grooming, total arm entries, rearing count, grooming count, and head-dip count. Color intensity represents the strength and direction of correlations (red, positive; blue, negative). Across cohorts, lipid–behavior associations exhibit structured, partially block-like patterns rather than isolated pairwise correlations, indicating alignment between lipidomic configurations and low-dimensional behavioral organization, with cohort-specific differences in the relative weighting of individual lipid–behavior relationships.

When lipidomic and behavioral variables were analyzed jointly, structured patterns of association emerged, with regions of coordinated lipid–behavior covariance emerging across cohorts rather than uniformly distributed pairwise relationships (Figure 5A–D). This organization supports alignment between behavioral organization and coordinated plasma lipid states at the multivariate level, rather than being determined by individual lipid species in isolation. Because all behavioral assessments and plasma sampling were conducted within a consistent daytime window under stable environmental conditions, the observed lipid–behavior alignment is unlikely to be attributable to circadian or seasonal variation.

Furthermore, because all samples were collected under identical anesthesia and handling conditions, any procedure-related metabolic effects would be uniformly distributed across cohorts and are therefore unlikely to account for the observed lipidomic organization.

Consistent with FDR-adjusted analysis, no individual lipid–behavior correlations remained formally significant in any cohort; accordingly, these associations were not interpreted as independent statistical evidence. Instead, emphasis was placed on the persistence of moderate-to-strong effect sizes organized into coherent patterns that aligned with lipidomic modules and behavioral axes, supporting a reproducible state-level correspondence across cohorts.

Accordingly, interpretation focuses on alignment between lipidomic states and behavioral organization rather than on the directionality of individual lipid–behavior correlations.

In Cohort A, a lipid configuration enriched in longer-chain unsaturated components, including the C18-associated fraction, aligned with higher exploratory engagement and earlier grooming initiation, whereas an opposing shorter-chain-weighted configuration showed weaker or inverse alignment with these behavioral features (Figure 5A). In Cohort B, grooming initiation again showed prominent alignment with lipidomic state, although the behavioral directionality of individual C18-associated lipids differed from Cohort A, indicating that lipid–behavior associations are context-dependent within a preserved multivariate structure (Figure 5B). In Cohort C, grooming initiation and exploratory measures aligned with partially distinct lipid subsets, with the shorter-chain/saturated versus C18-oriented contrast remaining apparent at the state level but less cleanly resolved at the level of individual lipid–behavior pairings (Figure 5C). In Cohort D, a dominant lipid module with strong contribution from the C18-associated fraction aligned with a combined behavioral engagement dimension encompassing grooming and investigatory activity, rather than with arm-entry-driven exploration (Figure 5D). Across cohorts, the most consistent feature was therefore not the behavior of any single lipid, but the alignment between plasma lipid states and behavioral dimensions, indicating a state-level correspondence between metabolic organization and spontaneous behavior.

Across cohorts, lipid–behavior associations forming coherent blocks typically showed moderate-to-strong effect sizes (|r| commonly ~0.4–0.8), indicating coordinated variation within lipid–behavior modules, whereas isolated lipid–behavior pairs outside these blocks were comparatively weak. Consistent with FDR-adjusted analysis, these patterns are interpreted at the level of multivariate organization rather than as independent pairwise associations. Complete lipid–behavior correlation matrices for all cohorts are provided in Supplementary Tables S4A–S4D.

Cohort-dependent directionality of individual lipid–behavior correlations reflects the multivariate organization of the underlying system rather than inconsistency. As detailed by Quinn et al. (2019), lipidomic variables are compositional and therefore mathematically interdependent, such that the direction and magnitude of any single correlation depend on the overall covariance structure of the dataset rather than representing a fixed pairwise property [11]. In our study, this is directly relevant because each lipid variable was analyzed as a proportion of the total identified plasma fatty-acid pool; accordingly, modest cohort-specific redistribution between the C18-centered and C14/C16-centered modules can change the apparent sign of an individual lipid–behavior correlation even when the higher-order state structure remains preserved. Because the behavioral measures are also correlated and load differently across cohorts onto shared exploratory and grooming-related dimensions, individual pairwise associations are expected to vary more than the underlying lipid-state–behavior alignment.

Consistent with this, Greenacre et al. (2021) showed that in high-dimensional compositional datasets, the underlying log-ratio geometry and low-dimensional structure are preserved even when individual variable representations or pairwise relationships vary across datasets [12]. In the present study, this implies that a modest cohort-specific redistribution of relative fatty-acid abundances can alter the sign of individual lipid–behavior correlations, while the global organization of lipidomic states and their alignment with behavioral dimensions remains stable [12].

Importantly, the lipid variables analyzed here represent compositional measures, and any pathway-level interpretation (e.g., desaturation or elongation processes) reflects indirect inference from fatty-acid relationships rather than the direct quantification of enzymatic activity.

This statistical interpretation is biologically plausible. Levental et al. (2020) demonstrated that lipid systems are maintained through coordinated compensatory remodeling, whereby changes in one part of the lipidome are balanced by opposing shifts in other lipid classes to preserve functional homeostasis [28]. Similarly, Cho et al. (2023) described lipid metabolism as a dynamic remodeling network in which multiple lipid configurations can represent stable physiological states [29], and Li et al. (2026) further emphasized that the functional significance of individual lipid species is inherently context-dependent within broader metabolic and signaling environments [30]. Thus, even when animals are matched for strain, age, sex, weight, diet, and housing, intrinsic variation in steady-state metabolic regulation can produce distinct but physiologically normal lipidomic configurations across independent cohorts. Within this framework, the reproducible feature is not the direction of each individual lipid–behavior correlation, but the preservation of structured, low-dimensional alignment between plasma lipid states and behavioral organization. Accordingly, interpretation is focused on this stable state-level correspondence rather than on the precise sign of isolated pairwise associations.

While cohort-dependent differences in lipid weighting were observed, several features argue against technical batch effects as the primary driver. First, all cohorts were processed under identical experimental and analytical conditions, reducing the likelihood of systematic analytical bias. Second, lipid–lipid correlation matrices within each cohort exhibited coherent modular organization rather than patterns expected from random technical variation. Third, PCA consistently resolved structured lipidomic states without evidence of global shifts indicative of analytical drift. Finally, the alignment between lipidomic states and behavioral organization was preserved across independent cohorts. Together, these observations support the interpretation that cohort-dependent differences primarily reflect biological variability within physiologically normal lipidomic states rather than technical artifacts.

### 3.6. Pathway-Level Interpretation of Lipidomic States

All pathway-level references (e.g., SCD1, ELOVL, FADS1/2) represent indirect biochemical inferences derived from fatty-acid compositional patterns and established metabolic pathway knowledge and do not reflect the direct measurement of enzyme expression, activity, or metabolic flux in the present study.

Integration of the observed plasma lipid composition covariance patterns with established lipid-metabolic pathway architecture supports the interpretation that the cohort-dependent lipid states identified here arise from coordinated regulation across hierarchically organized lipid remodeling axes rather than from isolated variation in individual fatty acids [31]. The consistent structure of lipid–lipid covariance, PCA-based stratification, and module-level opposition observed across cohorts aligns with stepwise modulation of unsaturation balance, carbon-chain allocation, long-chain PUFA maturation, and organization of PUFA substrate pools, without implying pathway activation or directional causality.

At the base of this hierarchy is a regulated balance between saturated and monounsaturated fatty acids. Monounsaturated species, most prominently palmitoleic acid (C16:1 n-7) and, where present, oleic acid (C18:1 n-9), consistently formed coherent lipid modules that contributed strongly to between-animal separation rather than fluctuating independently. Introduction of a single cis double bond alters acyl-chain packing and membrane order, and coordinated enrichment of these species is therefore consistent with a shift toward more fluid lipid assemblies at the systemic level [32]. The structured behavior of monounsaturated species relative to their saturated precursors supports regulated Δ9 desaturation as a steady-state property of the circulating lipid pool, consistent with differential allocation between rigid and fluidizing fatty-acid species [33].

Independent of unsaturation, lipidomic states were consistently differentiated by carbon-chain length. Fatty acids centered on 16-carbon backbones and those centered on 18-carbon backbones segregated across cohorts and did not vary in parallel, indicating selective routing of fatty-acid carbon rather than passive accumulation. At the pathway level, such non-parallel behavior is most consistently accounted for by regulated elongation of C16 substrates toward C18 backbones, classically mediated by elongation of very-long-chain fatty acid (ELOVL) family enzymes [34]. This routing reflects differential allocation between shorter-chain, metabolically labile fatty acids and longer-chain species that are preferentially incorporated into more stable lipid assemblies and serve as substrates for downstream desaturation and PUFA-related pathways.

Within this elongation-structured framework, longer-chain unsaturated species aligned with defined lipid modules rather than appearing as weak or diffuse intermediates, indicating coordinated handling rather than sporadic accumulation. This pattern is consistent with the stabilization of elongated fatty acids into structurally integrated lipid pools [35]. At the pathway level, such stabilization is directly consistent with terminal desaturation of elongated substrates, a role classically attributed to fatty acid desaturase 1 (FADS1) [36]. Although enzymatic activity was not directly measured in the present study, the coherent behavior of long-chain unsaturated species supports the regulated completion of PUFA structure as part of stable lipidomic states rather than transient metabolic flux.

Finally, variation involving polyunsaturated fatty acids is best interpreted in terms of the upstream organization of PUFA substrates rather than the activation of downstream signaling pathways [37]. Fatty acid desaturase 2 (FADS2) occupies a central position in networks governing PUFA synthesis and phospholipid remodeling, positioning it as a determinant of how PUFA species are incorporated into circulating lipid pools that may later serve as signaling substrates. In the present study, cohort-specific differences in PUFA-containing lipid modules therefore reflect structured differences in PUFA substrate architecture within the plasma lipidome, without implying phospholipase activation, eicosanoid production, or inflammatory signaling.

**Figure 6:**
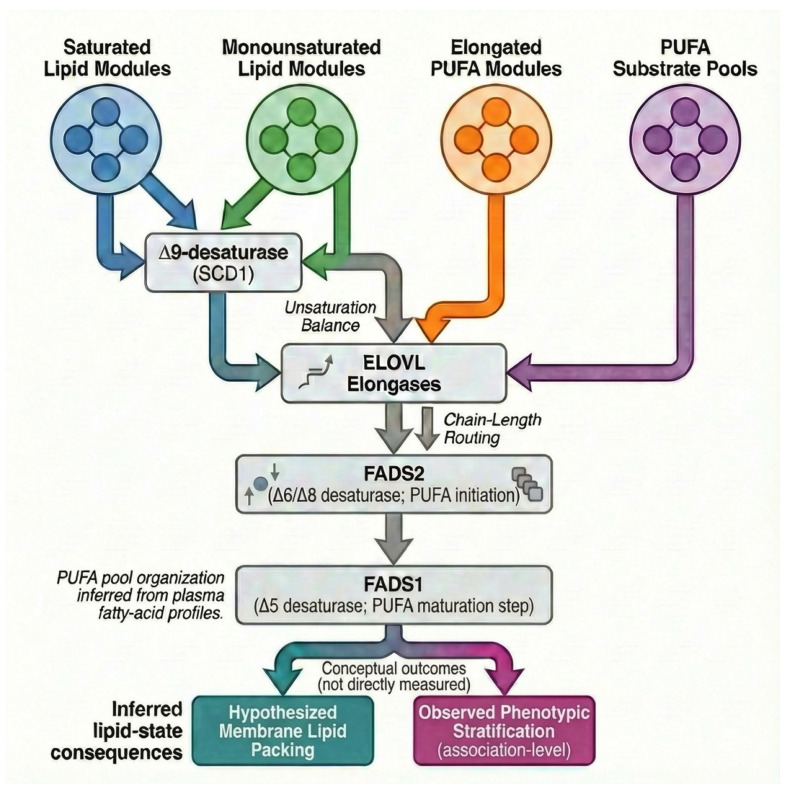
Conceptual model of hierarchical lipidomic organization. Schematic representation of inferred lipid-regulatory layers linking desaturation, elongation, and remodeling processes, derived from fatty-acid compositional relationships; no direct measurements of enzyme activity or pathway flux were performed. Downstream elements, including hypothesized membrane lipid packing effects and observed phenotypic stratification (association-level), represent conceptual interpretations derived from the observed lipidomic organization rather than experimentally validated outcomes.

Collectively, these findings support a hierarchical lipid-regulation model in which Δ9 desaturation establishes unsaturation balance, ELOVL-mediated elongation determines chain-length allocation, FADS1-associated terminal desaturation consolidates long-chain PUFA structure, and phospholipid remodeling organizes PUFA substrate pools. This framework provides a mechanistically coherent interpretation of the consistent lipidomic states observed across cohorts and their systematic alignment with low-dimensional behavioral organization, fully supported by plasma fatty-acid profiling. The hierarchical organization of these inferred lipidomic control points and their systemic consequences is schematically summarized in Figure 6.

## 4. Limitations and Future Directions

This study was intentionally designed to characterize baseline metabolic–behavioral organization under minimally perturbed physiological conditions, with an emphasis on state-level structure rather than mechanistic perturbation or causal testing. Accordingly, the analytical framework prioritizes reproducible covariance patterns and organizational principles in healthy animals, providing a foundation for more targeted mechanistic and intervention-based studies.

The present study defines a pathway-anchored framework for interpreting plasma lipidomic organization and its integration with experimentally measured behavioral phenotypes. While the current analyses establish stable, cohort-dependent lipidomic states with strong internal consistency, several extensions will further refine and validate the inferred regulatory architecture.

Lipid-metabolic control points involving Δ9 desaturation, carbon-chain elongation, terminal desaturation, and phospholipid remodeling are inferred from coherent lipid-module behavior and pathway congruence rather than direct molecular quantification. Future integration with targeted transcriptomic or proteomic profiling will enable direct testing of these regulatory layers and their quantitative contribution to systemic lipid states.

Incorporation of stable-isotope tracing represents a critical next step to resolve carbon routing and desaturation flux underlying the non-parallel behavior of C16- and C18-centered fatty-acid pools. Such approaches would distinguish altered synthesis, elongation efficiency, and turnover as drivers of lipidomic stratification.

Although plasma lipidomics captures integrated organism-level regulation, extension to tissue-resolved lipidomic analyses would clarify the organ-specific origins of the circulating lipid modules identified here. Longitudinal designs combining lipidomic profiling with repeated behavioral testing would further define temporal stability and potential predictive relationships between systemic lipid organization and behavioral output.

Finally, application of this hierarchical, module-based framework to human plasma lipidomic datasets will determine whether comparable regulatory architectures operate across species, providing a foundation for translational stratification and hypothesis-driven validation without presupposing disease-specific signaling activation.

## 5. Conclusions

This study shows that baseline plasma fatty-acid profiles in healthy rats are organized into reproducible, low-dimensional lipidomic states rather than varying randomly across individuals. Across four independent cohorts, correlation-based and multivariate analyses consistently revealed modular lipid organization dominated by coordinated shifts between C14–C16-centered and C18-centered fatty-acid species. Behavioral measures likewise resolved into constrained exploratory and grooming-related dimensions.

Integration of lipidomic and behavioral data demonstrated that associations occur primarily at the state level, with coherent lipid modules aligning with low-dimensional behavioral organization despite cohort-dependent variability in individual lipid–behavior relationships. While the directionality of individual correlations varied across cohorts, the alignment between fatty-acid-defined plasma lipid states and behavioral dimensions remained preserved, indicating robust metabolic–behavioral coupling under physiological conditions.

The interpretation of lipid–behavior relationships in the present study was undertaken with consideration of potential confounding factors. Key experimental conditions, including housing environment, microbiome-related conditions, testing time, and sampling procedures, were standardized as part of the study design to reduce variability across cohorts. At the same time, given the complexity of biological systems, contributions from microbiome variability, circadian influences, or sampling-related effects cannot be fully excluded. Accordingly, the observed associations are interpreted conservatively as reflecting reproducible multivariate organization rather than definitive causal relationships between specific lipid species and behavioral outcomes.

The principal contribution of this work is the establishment of a correlation-structure-based, state-level framework for interpreting plasma lipidomics and its behavioral relevance in healthy animals, without inferring causality or pathway activation. This modular, pathway-informed perspective provides a foundation for future mechanistic and translational studies aimed at resolving how systemic lipid metabolism contributes to phenotypic variability in health and disease.

These interpretations are derived from compositional lipidomic data and should be understood as pathway-informed inferences rather than direct measurements of enzymatic activity.

## Figures and Tables

**Table 1:** Identified plasma fatty acid methyl esters (FAMEs) consistently detected across all cohorts. Compound identities were assigned by dual-column GC-MS retention behavior and NIST EI spectral matching, with verification against the Supelco 37-component FAME reference standard.

Peak	RT (min)	FAME Candidate
1	12.493	Tridecanoic Acid Methyl Ester (C13:0)
2	12.913	Myristic Acid Methyl Ester (C14:0)
3	13.632	Unresolved feature in the C14 region
4	14.164	Putative C14:1-associated feature
5	16.332	Palmitoleic Acid Methyl Ester (C16:1 n7)
6	16.888	cis-10-Heptadecenoic Acid Methyl Ester (C17:1 n7)
7	17.278	Stearic Acid Methyl Ester (C18:0)
8	17.420	Oleic Acid Methyl Ester (C18:1 n9)
9	19.917	γ-Linolenic Acid Methyl Ester (C18:3 n6)

## Data Availability

The data supporting the findings of this study are available within the article and its Supplementary Materials.

## Supplementary Materials

The following supporting information can be downloaded at: https://www.mdpi.com/article/10.3390/biology15080619/s1, Supplementary Tables S1A–S1D provide raw behavioral observations for all animals, while Supplementary Tables S2–S4 present the corresponding lipid–lipid, behavior–behavior, and lipid–behavior correlation matrices (Pearson r) together with Benjamini–Hochberg false discovery rate (FDR)-adjusted *p*-values (q-values), which were used to characterize multivariate organization and integration patterns across cohorts. Figure S1. Principal component analysis (PCA) of plasma lipidomic profiles following variance scaling. PCA was repeated using standardized lipid variables (centered and variance-scaled) for all cohorts (A–D). Panels A, B, and D are visually identical to the corresponding plots in Figure 3. In contrast, Cohort C shows a modest change in cluster geometry, while overall group separation remains preserved.

## Abbreviations

Abbreviation	Full Term
AR	Analytical Reagent
AUC	Area under the Curve
BF_3_	Boron Trifluoride
EI	Electron Ionization
ELOVL	Elongation of Very-Long-Chain Fatty Acids
FADS1	Fatty Acid Desaturase 1
FADS2	Fatty Acid Desaturase 2
FAME	Fatty Acid Methyl Ester
FAME-37	37-Component Fatty Acid Methyl Ester Reference Mixture (Supelco)
FELASA	Federation of European Laboratory Animal Science Associations
FDR	False Discovery Rate
GC–FID	Gas Chromatography–Flame Ionization Detection
GC–MS	Gas Chromatography–Mass Spectrometry
HPLC	High-Performance Liquid Chromatography
IACUC	Institutional Animal Care and Use Committee
KOH	Potassium Hydroxide
MS	Mass Spectrometry
NIST	National Institute of Standards and Technology
PC	Principal Component
PC1	Principal Component 1
PC2	Principal Component 2
PCA	Principal Component Analysis
PUFA	Polyunsaturated Fatty Acid
RT	Retention Time
SCD1	Stearoyl-CoA Desaturase 1
TIC	Total Ion Current
UPPC	University of Petra Pharmaceutical Center
