# Supplementary material for: State-Level Organization of Spontaneous Behavior and Its Association with Plasma Fatty-Acid Profiles in Healthy Rats
Source: Biology (Basel). 2026 Apr 16;15(8):619. doi: 10.3390/biology15080619 (PMC13112989; doi:10.3390/biology15080619)
Supplement: Supplementary file 1 [file biology-15-00619-s001.zip › biology-4204559-supplementary.pdf]

Supplementary Table S1A. Raw behavioral measurements for individual animals across cohort A.

Rat_ID	Lat_First_Groom (s)	Total_Arm_Entries	Rearing_Count	Grooming_Count	Head_Dip_Count
**Rat1**	300	18	4	1	11
**Rat2**	120	18	0	2	11
**Rat3**	90	12	0	4	5
**Rat4**	270	6	0	1	6
**Rat5**	60	7	0	2	7
**Rat6**	240	13	2	1	4
**Rat7**	180	18	3	1	14
**Rat8**	120	13	8	5	13

Supplementary Table S1B. Raw behavioral measurements for individual animals across cohort B.

Rat_ID	Lat_First_Groom (s)	Total_Arm_Entries	Rearing_Count	Grooming_Count	Head_Dip_Count
**Rat1**	142	15	8	1	9
**Rat2**	300	16	0	0	9
**Rat3**	45	18	0	4	13
**Rat4**	60	16	2	2	9
**Rat5**	90	7	3	1	6
**Rat6**	60	7	2	6	7
**Rat7**	300	3	0	0	1

Supplementary Table S1C. Raw behavioral measurements for individual animals across cohort C.

Rat_ID	Lat_First_Groom (s)	Total_Arm_Entries	Rearing_Count	Grooming_Count	Head_Dip_Count
**Rat1**	240	10	11	1	23
**Rat2**	150	15	13	3	19
**Rat3**	120	18	4	3	20
**Rat4**	300	20	5	0	14
**Rat5**	40	12	1	3	8
**Rat6**	90	8	2	3	6
**Rat7**	145	10	2	2	12

Supplementary Table S1D. Raw behavioral measurements for individual animals across cohort D.

Rat_ID	Lat_First_Groom (s)	Total_Arm_Entries	Rearing_Count	Grooming_Count	Head_Dip_Count
**Rat1**	50	7	3	2	10
**Rat2**	165	12	1	1	4
**Rat3**	210	8	1	6	12
**Rat4**	90	1	0	2	6
**Rat5**	120	7	0	2	6
**Rat6**	300	4	0	0	1
**Rat7**	60	7	2	2	7
**Rat8**	45	8	0	2	11

Supplementary Table S2A. Lipid–lipid correlation matrix for Cohort A. Pearson correlation coefficients (r) among the nine plasma lipid variables (Lipids 1–9) measured in Cohort A (A1–A8). Values were computed from proportional lipid abundances (% of total identified lipids). Corresponding Benjamini–Hochberg false discovery rate (FDR)-adjusted p-values (q-values) are provided. Correlations are presented to characterize coordinated variation within the plasma lipidome rather than as independent pairwise statistical associations.

	Lipid 1	Lipid 2	Lipid 3	Lipid 4	Lipid 5	Lipid 6	Lipid 7	Lipid 8	Lipid 9
Lipid 1	—	0.26 (0.870)	−0.10 (0.915)	0.38 (0.860)	−0.32 (0.860)	−0.51 (0.915)	0.66 (0.486)	−0.28 (0.860)	−0.44 (0.831)
Lipid 2	0.26 (0.870)	—	0.66 (0.486)	0.03 (0.787)	0.10 (0.831)	0.13 (0.915)	−0.24 (0.870)	−0.76 (0.486)	−0.53 (0.787)
Lipid 3	−0.10 (0.915)	0.66 (0.486)	—	−0.20 (0.486)	−0.31 (0.486)	0.12 (0.870)	−0.01 (0.981)	−0.80 (0.486)	−0.43 (0.831)
Lipid 4	0.38 (0.860)	0.03 (0.787)	−0.20 (0.486)	—	−0.29 (0.860)	0.30 (0.831)	0.05 (0.960)	−0.37 (0.860)	−0.42 (0.831)
Lipid 5	−0.32 (0.860)	0.10 (0.831)	−0.31 (0.486)	−0.29 (0.860)	—	0.11 (0.870)	−0.71 (0.486)	0.47 (0.831)	0.21 (0.870)
Lipid 6	−0.51 (0.915)	0.13 (0.915)	0.12 (0.870)	0.30 (0.831)	0.11 (0.870)	—	−0.58 (0.678)	−0.19 (0.870)	−0.35 (0.860)
Lipid 7	0.66 (0.486)	−0.24 (0.870)	−0.01 (0.981)	0.05 (0.960)	−0.71 (0.486)	−0.58 (0.678)	—	−0.08 (0.928)	−0.21 (0.870)
Lipid 8	−0.28 (0.860)	−0.76 (0.486)	−0.80 (0.486)	−0.37 (0.860)	0.47 (0.831)	−0.19 (0.870)	−0.08 (0.928)	—	0.65 (0.486)
Lipid 9	−0.44 (0.831)	−0.53 (0.787)	−0.43 (0.831)	−0.42 (0.831)	0.21 (0.870)	−0.35 (0.860)	−0.21 (0.870)	0.65 (0.486)	—

Supplementary Table S2B. Lipid–lipid correlation matrix for Cohort B. Pearson correlation coefficients (r) among the nine plasma lipid variables (Lipids 1–9) measured in Cohort B (B1–B8). Values were computed from proportional lipid abundances (% of total identified lipids). Corresponding Benjamini–Hochberg false discovery rate (FDR)-adjusted p-values (q-values) are provided. Correlations are presented to characterize coordinated variation within the plasma lipidome rather than as independent pairwise statistical associations.

	Lipid 1	Lipid 2	Lipid 3	Lipid 4	Lipid 5	Lipid 6	Lipid 7	Lipid 8	Lipid 9
Lipid 1	—	−0.25 (0.922)	0.71 (0.212)	−0.79 (0.137)	−0.62 (0.322)	−0.75 (0.186)	0.27 (0.922)	−0.93 (0.033)	−0.84 (0.089)
Lipid 2	−0.25 (0.922)	—	−0.41 (0.665)	0.44 (0.632)	−0.31 (0.817)	−0.22 (0.922)	0.18 (0.922)	0.57 (0.365)	0.54 (0.402)
Lipid 3	0.71 (0.212)	−0.41 (0.665)	—	−0.65 (0.271)	−0.60 (0.322)	−0.69 (0.239)	−0.24 (0.922)	−0.78 (0.137)	−0.78 (0.137)
Lipid 4	−0.79 (0.137)	0.44 (0.632)	−0.65 (0.271)	—	0.14 (0.922)	0.61 (0.322)	−0.48 (0.602)	0.81 (0.122)	0.62 (0.322)
Lipid 5	−0.62 (0.322)	−0.31 (0.817)	−0.60 (0.322)	0.14 (0.922)	—	0.74 (0.186)	0.26 (0.922)	0.43 (0.665)	0.49 (0.602)
Lipid 6	−0.75 (0.186)	−0.22 (0.922)	−0.69 (0.239)	0.61 (0.322)	0.74 (0.186)	—	−0.30 (0.817)	0.56 (0.365)	0.51 (0.402)
Lipid 7	0.27 (0.922)	0.18 (0.922)	−0.24 (0.922)	−0.48 (0.602)	0.26 (0.922)	−0.30 (0.817)	—	−0.14 (0.922)	0.04 (0.992)
Lipid 8	−0.93 (0.033)	0.57 (0.365)	−0.78 (0.137)	0.81 (0.122)	0.43 (0.665)	0.56 (0.365)	−0.14 (0.922)	—	0.94 (0.033)
Lipid 9	−0.84 (0.089)	0.54 (0.402)	−0.78 (0.137)	0.62 (0.322)	0.49 (0.602)	0.51 (0.402)	0.04 (0.992)	0.94 (0.033)	—

Supplementary Table S2C. Lipid–lipid correlation matrix for Cohort C. Pearson correlation coefficients (r) among the nine plasma lipid variables (Lipids 1–9) measured in Cohort C (C1–C8). Values were computed from proportional lipid abundances (% of total identified lipids). Corresponding Benjamini–Hochberg false discovery rate (FDR)-adjusted p-values (q-values) are provided. Correlations are presented to characterize coordinated variation within the plasma lipidome rather than as independent pairwise statistical associations.

	Lipid 1	Lipid 2	Lipid 3	Lipid 4	Lipid 5	Lipid 6	Lipid 7	Lipid 8	Lipid 9
Lipid 1	—	−0.44 (0.602)	0.08 (0.992)	0.82 (0.122)	−0.45 (0.602)	−0.68 (0.239)	0.50 (0.486)	0.36 (0.665)	0.03 (0.992)
Lipid 2	−0.44 (0.602)	—	0.31 (0.817)	−0.21 (0.922)	−0.30 (0.817)	−0.07 (0.992)	−0.35 (0.665)	0.01 (0.992)	−0.28 (0.817)
Lipid 3	0.08 (0.992)	0.31 (0.817)	—	0.21 (0.922)	−0.88 (0.089)	−0.47 (0.602)	0.15 (0.922)	−0.47 (0.602)	−0.68 (0.239)
Lipid 4	0.82 (0.122)	−0.21 (0.922)	0.21 (0.922)	—	−0.52 (0.486)	−0.57 (0.402)	0.11 (0.992)	0.55 (0.402)	−0.09 (0.992)
Lipid 5	−0.45 (0.602)	−0.30 (0.817)	−0.88 (0.089)	−0.52 (0.486)	—	0.67 (0.239)	−0.32 (0.817)	0.22 (0.922)	0.68 (0.239)
Lipid 6	−0.68 (0.239)	−0.07 (0.992)	−0.47 (0.602)	−0.57 (0.402)	0.67 (0.239)	—	−0.46 (0.602)	−0.17 (0.922)	−0.07 (0.992)
Lipid 7	0.50 (0.486)	−0.35 (0.665)	0.15 (0.922)	0.11 (0.992)	−0.32 (0.817)	−0.46 (0.602)	—	−0.46 (0.602)	−0.17 (0.922)
Lipid 8	0.36 (0.665)	0.01 (0.992)	−0.47 (0.602)	0.55 (0.402)	0.22 (0.922)	−0.17 (0.922)	−0.46 (0.602)	—	0.58 (0.365)
Lipid 9	0.03 (0.992)	−0.28 (0.817)	−0.68 (0.239)	−0.09 (0.992)	0.68 (0.239)	−0.07 (0.992)	−0.17 (0.922)	0.58 (0.365)	—

Supplementary Table S2D. Lipid–lipid correlation matrix for Cohort D. Pearson correlation coefficients (r) among the nine plasma lipid variables (Lipids 1–9) measured in Cohort D (D1–D8). Values were computed from proportional lipid abundances (% of total identified lipids). Corresponding Benjamini–Hochberg false discovery rate (FDR)-adjusted p-values (q-values) are provided. Correlations are presented to characterize coordinated variation within the plasma lipidome rather than as independent pairwise statistical associations.

	Lipid 1	Lipid 2	Lipid 3	Lipid 4	Lipid 5	Lipid 6	Lipid 7	Lipid 8	Lipid 9
Lipid 1	—	−0.30 (0.860)	−0.24 (0.870)	−0.38 (0.831)	−0.19 (0.915)	0.24 (0.870)	−0.30 (0.860)	−0.32 (0.860)	−0.29 (0.860)
Lipid 2	−0.30 (0.860)	—	−0.04 (0.992)	−0.45 (0.831)	0.26 (0.870)	−0.44 (0.831)	−0.39 (0.831)	−0.39 (0.831)	−0.38 (0.831)
Lipid 3	−0.24 (0.870)	−0.04 (0.992)	—	−0.46 (0.831)	−0.64 (0.678)	−0.22 (0.870)	−0.60 (0.678)	−0.55 (0.678)	−0.58 (0.678)
Lipid 4	−0.38 (0.831)	−0.45 (0.831)	−0.46 (0.831)	—	0.42 (0.831)	0.03 (0.992)	0.98 (0.001)	0.93 (0.003)	0.98 (0.001)
Lipid 5	−0.19 (0.915)	0.26 (0.870)	−0.64 (0.678)	0.42 (0.831)	—	−0.31 (0.860)	0.46 (0.831)	0.50 (0.831)	0.47 (0.831)
Lipid 6	0.24 (0.870)	−0.44 (0.831)	−0.22 (0.870)	0.03 (0.992)	−0.31 (0.860)	—	0.06 (0.992)	0.01 (0.992)	−0.01 (0.992)
Lipid 7	−0.30 (0.860)	−0.39 (0.831)	−0.60 (0.678)	0.98 (0.001)	0.46 (0.831)	0.06 (0.992)	—	0.96 (0.001)	0.99 (0.001)
Lipid 8	−0.32 (0.860)	−0.39 (0.831)	−0.55 (0.678)	0.93 (0.003)	0.50 (0.831)	0.01 (0.992)	0.96 (0.001)	—	0.93 (0.003)
Lipid 9	−0.29 (0.860)	−0.38 (0.831)	−0.58 (0.678)	0.98 (0.001)	0.47 (0.831)	−0.01 (0.992)	0.99 (0.001)	0.93 (0.003)	—

Supplementary Table S3A. Behavioral correlation matrix for Cohort A (Y-maze variables). Pearson correlation coefficients (r) among behavioral variables measured during the Y-maze task in Cohort A (A1–A8), including latency to first grooming, total arm entries, rearing count, grooming count, and head-dip count. Corresponding Benjamini–Hochberg false discovery rate (FDR)-adjusted p-values (q-values) are provided. Correlations are presented to characterize low-dimensional behavioral organization rather than as independent pairwise statistical associations.

	**Latency**	**Arm**	**Rearing**	**Groom**	**Head**
Latency	—	0.16 (0.915)	0.14 (0.915)	−0.64 (0.486)	−0.04 (0.981)
Arm	0.16 (0.915)	—	0.34 (0.860)	−0.10 (0.915)	0.64 (0.486)
Rearing	0.14 (0.915)	0.34 (0.860)	—	0.42 (0.831)	0.62 (0.486)
Groom	−0.64 (0.486)	−0.10 (0.915)	0.42 (0.831)	—	0.12 (0.915)
Head	−0.04 (0.981)	0.64 (0.486)	0.62 (0.486)	0.12 (0.915)	—

Supplementary Table S3B. Behavioral correlation matrix for Cohort B (Y-maze variables). Pearson correlation coefficients (r) among behavioral variables measured during the Y-maze task in Cohort B (B1–B8), including latency to first grooming, total arm entries, rearing count, grooming count, and head-dip count. Corresponding Benjamini–Hochberg false discovery rate (FDR)-adjusted p-values (q-values) are provided. Correlations are presented to characterize low-dimensional behavioral organization rather than as independent pairwise statistical associations.

	**Latency**	**Arm**	**Rearing**	**Groom**	**Head**
Latency	—	−0.52 (0.402)	0.61 (0.322)	−0.68 (0.239)	−0.61 (0.322)
Arm	−0.52 (0.402)	—	−0.16 (0.922)	0.31 (0.817)	0.91 (0.033)
Rearing	0.61 (0.322)	−0.16 (0.922)	—	−0.28 (0.817)	0.07 (0.992)
Groom	−0.68 (0.239)	0.31 (0.817)	−0.28 (0.817)	—	0.57 (0.365)
Head	−0.61 (0.322)	0.91 (0.033)	0.07 (0.992)	0.57 (0.365)	—

Supplementary Table S3C. Behavioral correlation matrix for Cohort C (Y-maze variables). Pearson correlation coefficients (r) among behavioral variables measured during the Y-maze task in Cohort C (C1–C8), including latency to first grooming, total arm entries, rearing count, grooming count, and head-dip count. Corresponding Benjamini–Hochberg false discovery rate (FDR)-adjusted p-values (q-values) are provided. Correlations are presented to characterize low-dimensional behavioral organization rather than as independent pairwise statistical associations.

	**Latency**	**Arm**	**Rearing**	**Groom**	**Head**
Latency	—	0.18 (0.922)	0.62 (0.322)	−0.75 (0.186)	0.71 (0.212)
Arm	0.18 (0.922)	—	0.31 (0.817)	−0.51 (0.402)	0.58 (0.365)
Rearing	0.62 (0.322)	0.31 (0.817)	—	−0.19 (0.922)	0.84 (0.089)
Groom	−0.75 (0.186)	−0.51 (0.402)	−0.19 (0.922)	—	−0.34 (0.665)
Head	0.71 (0.212)	0.58 (0.365)	0.84 (0.089)	−0.34 (0.665)	—

Supplementary Table S3D. Behavioral correlation matrix for Cohort D (Y-maze variables). Pearson correlation coefficients (r) among behavioral variables measured during the Y-maze task in Cohort D (D1–D8), including latency to first grooming, total arm entries, rearing count, grooming count, and head-dip count. Corresponding Benjamini–Hochberg false discovery rate (FDR)-adjusted p-values (q-values) are provided. Correlations are presented to characterize low-dimensional behavioral organization rather than as independent pairwise statistical associations.

	Latency	Arm	Rearing	Groom	Head
Latency	—	−0.30 (0.860)	−0.38 (0.831)	−0.29 (0.860)	−0.71 (0.486)
Arm	−0.30 (0.860)	—	0.45 (0.831)	0.03 (0.992)	0.35 (0.860)
Rearing	−0.38 (0.831)	0.45 (0.831)	—	0.21 (0.870)	0.56 (0.678)
Groom	−0.29 (0.860)	0.03 (0.992)	0.21 (0.870)	—	0.47 (0.831)
Head	−0.71 (0.486)	0.35 (0.860)	0.56 (0.678)	0.47 (0.831)	—

Supplementary Table S4A. Integrated lipid–behavior correlation matrix for Cohort A. Pearson correlation coefficients (r) between plasma lipid variables (Lipids 1–9) and behavioral measures in Cohort A (A1–A8). Behavioral variables include latency to first grooming, total arm entries, rearing count, grooming count, and head-dip count. Corresponding Benjamini–Hochberg false discovery rate (FDR)-adjusted p-values (q-values) are provided. Correlations are presented to characterize the alignment between lipidomic and behavioral organization at the multivariate level rather than as independent pairwise statistical associations.

Lipid	Latency	Arm	Rearing	Groom	Head
Lipid 1	−0.08 (0.981)	−0.29 (0.860)	0.40 (0.831)	−0.03 (0.992)	0.01 (0.992)
Lipid 2	0.53 (0.787)	−0.30 (0.860)	0.07 (0.992)	−0.21 (0.915)	−0.50 (0.831)
Lipid 3	0.15 (0.915)	−0.25 (0.870)	0.33 (0.860)	0.46 (0.831)	−0.16 (0.915)
Lipid 4	−0.03 (0.992)	−0.83 (0.486)	−0.37 (0.860)	−0.28 (0.860)	−0.34 (0.860)
Lipid 5	0.03 (0.992)	0.28 (0.860)	−0.57 (0.678)	−0.41 (0.831)	−0.36 (0.860)
Lipid 6	0.56 (0.787)	−0.23 (0.870)	−0.30 (0.860)	−0.38 (0.860)	−0.17 (0.915)
Lipid 7	−0.33 (0.860)	−0.03 (0.992)	0.75 (0.486)	0.54 (0.787)	0.43 (0.831)
Lipid 8	−0.26 (0.870)	0.67 (0.486)	−0.17 (0.915)	−0.16 (0.915)	0.39 (0.831)
Lipid 9	−0.23 (0.870)	0.56 (0.787)	−0.31 (0.860)	−0.10 (0.915)	0.25 (0.870)

Supplementary Table S4B. Integrated lipid–behavior correlation matrix for Cohort B. Pearson correlation coefficients (r) between plasma lipid variables (Lipids 1–9) and behavioral measures in Cohort B (B1–B8). Behavioral variables include latency to first grooming, total arm entries, rearing count, grooming count, and head-dip count. Corresponding Benjamini–Hochberg false discovery rate (FDR)-adjusted p-values (q-values) are provided. Correlations are presented to characterize the alignment between lipidomic and behavioral organization at the multivariate level rather than as independent pairwise statistical associations.

Lipid	Latency	Arm	Rearing	Groom	Head
Lipid 1	0.40 (0.665)	0.44 (0.632)	0.53 (0.402)	−0.36 (0.665)	0.28 (0.817)
Lipid 2	−0.14 (0.922)	−0.44 (0.632)	−0.10 (0.992)	0.33 (0.817)	−0.17 (0.922)
Lipid 3	0.66 (0.239)	0.44 (0.632)	−0.07 (0.992)	−0.65 (0.271)	0.20 (0.922)
Lipid 4	−0.73 (0.186)	0.01 (0.992)	−0.39 (0.665)	0.62 (0.322)	0.29 (0.817)
Lipid 5	−0.17 (0.922)	−0.50 (0.402)	0.01 (0.992)	0.03 (0.992)	−0.55 (0.365)
Lipid 6	−0.57 (0.365)	−0.01 (0.992)	−0.21 (0.922)	0.40 (0.665)	0.02 (0.992)
Lipid 7	0.11 (0.992)	−0.59 (0.365)	0.70 (0.212)	−0.08 (0.992)	−0.55 (0.365)
Lipid 8	−0.38 (0.665)	−0.56 (0.365)	−0.49 (0.602)	0.48 (0.602)	−0.31 (0.817)
Lipid 9	−0.23 (0.870)	−0.69 (0.239)	−0.48 (0.602)	0.52 (0.402)	−0.46 (0.632)

Supplementary Table S4C. Integrated lipid–behavior correlation matrix for Cohort C. Pearson correlation coefficients (r) between plasma lipid variables (Lipids 1–9) and behavioral measures in Cohort C (C1–C8). Behavioral variables include latency to first grooming, total arm entries, rearing count, grooming count, and head-dip count. Corresponding Benjamini–Hochberg false discovery rate (FDR)-adjusted p-values (q-values) are provided. Correlations are presented to characterize the alignment between lipidomic and behavioral organization at the multivariate level rather than as independent pairwise statistical associations.

Lipid	Latency	Arm	Rearing	Groom	Head
Lipid 1	−0.03 (0.992)	−0.07 (0.992)	−0.71 (0.212)	−0.17 (0.922)	−0.80 (0.137)
Lipid 2	0.38 (0.665)	−0.37 (0.665)	0.47 (0.602)	−0.39 (0.665)	0.53 (0.402)
Lipid 3	0.35 (0.665)	0.04 (0.992)	−0.26 (0.870)	−0.65 (0.271)	−0.05 (0.992)
Lipid 4	−0.27 (0.870)	−0.34 (0.665)	−0.63 (0.322)	−0.01 (0.992)	−0.89 (0.089)
Lipid 5	−0.39 (0.665)	0.18 (0.922)	0.37 (0.665)	0.71 (0.212)	0.31 (0.817)
Lipid 6	0.03 (0.992)	0.23 (0.870)	0.79 (0.137)	0.26 (0.870)	0.49 (0.602)
Lipid 7	0.07 (0.992)	−0.18 (0.922)	−0.52 (0.402)	−0.17 (0.922)	−0.37 (0.665)
Lipid 8	−0.39 (0.665)	−0.22 (0.870)	−0.16 (0.915)	0.40 (0.665)	−0.38 (0.665)
Lipid 9	−0.55 (0.365)	0.08 (0.992)	−0.25 (0.870)	0.68 (0.239)	−0.04 (0.992)

Supplementary Table S4D. Integrated lipid–behavior correlation matrix for Cohort D. Pearson correlation coefficients (r) between plasma lipid variables (Lipids 1–9) and behavioral measures in Cohort D (D1–D8). Behavioral variables include latency to first grooming, total arm entries, rearing count, grooming count, and head-dip count. Corresponding Benjamini–Hochberg false discovery rate (FDR)-adjusted p-values (q-values) are provided. Correlations are presented to characterize the alignment between lipidomic and behavioral organization at the multivariate level rather than as independent pairwise statistical associations.

Lipid	Latency	Arm	Rearing	Groom	Head
Lipid 1	0.42 (0.831)	0.27 (0.870)	0.03 (0.992)	0.85 (0.486)	0.45 (0.831)
Lipid 2	−0.16 (0.915)	−0.03 (0.992)	−0.17 (0.915)	−0.04 (0.992)	−0.04 (0.992)
Lipid 3	−0.34 (0.860)	0.13 (0.915)	0.79 (0.486)	−0.12 (0.915)	0.23 (0.870)
Lipid 4	0.11 (0.915)	−0.27 (0.870)	−0.31 (0.860)	−0.55 (0.678)	−0.50 (0.831)
Lipid 5	0.53 (0.787)	−0.35 (0.860)	−0.60 (0.678)	−0.40 (0.831)	−0.82 (0.486)
Lipid 6	−0.26 (0.870)	−0.12 (0.915)	−0.37 (0.860)	0.31 (0.860)	0.42 (0.831)
Lipid 7	0.14 (0.915)	−0.19 (0.915)	−0.42 (0.831)	−0.48 (0.831)	−0.47 (0.831)
Lipid 8	0.14 (0.915)	0.01 (0.992)	−0.35 (0.860)	−0.55 (0.678)	−0.56 (0.678)
Lipid 9	0.18 (0.915)	−0.25 (0.870)	−0.39 (0.860)	−0.47 (0.831)	−0.48 (0.831)

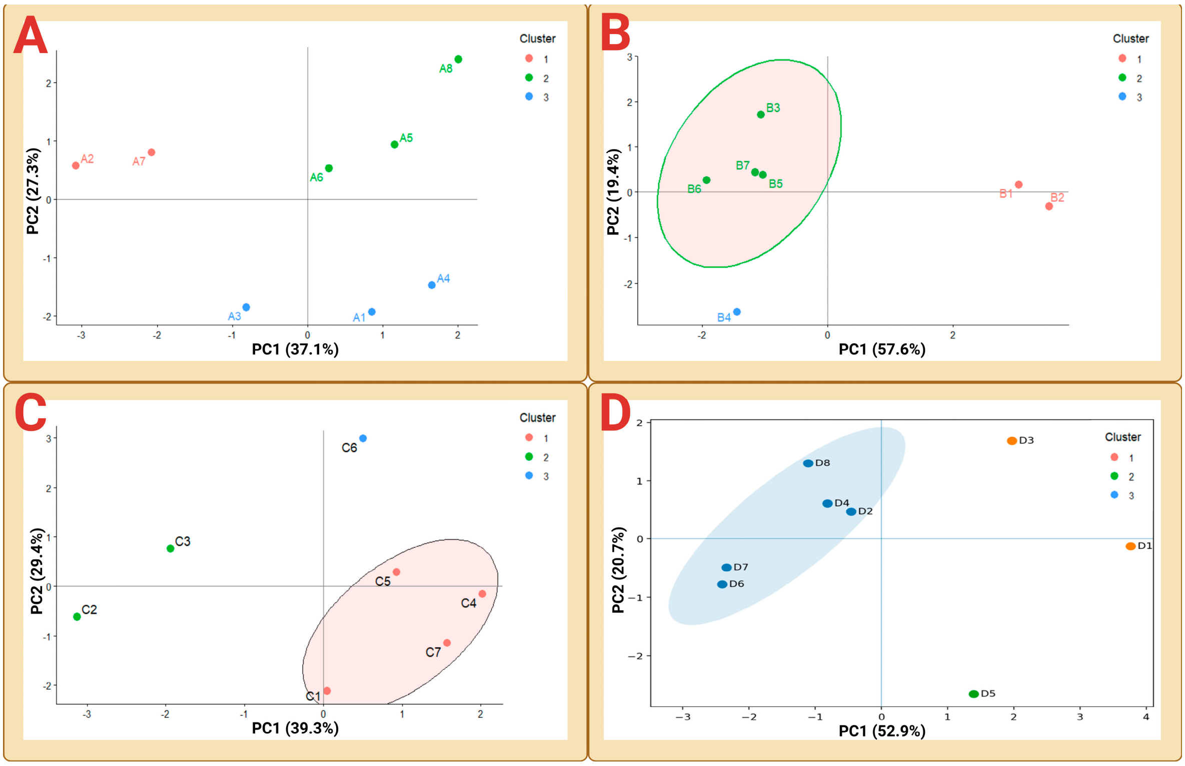

Figure S1. Principal component analysis (PCA) of plasma lipidomic profiles following variance scaling. PCA was repeated using standardized lipid variables (centered and variance-scaled) for all cohorts (A–D). Panels A, B, and D are visually identical to the corresponding plots in Figure 3. In contrast, Cohort C shows a modest change in cluster geometry, while overall group separation remains preserved.
